# Testosterone Pellet Associated Dermatitis: Report and Review of Testopel-related Cutaneous Adverse Effects

**DOI:** 10.7759/cureus.1560

**Published:** 2017-08-11

**Authors:** Lucas A Heldt Manica, Philip R Cohen

**Affiliations:** 1 John A. Burns School of Medicine, University of Hawaii Manoa; 2 Department of Dermatology, University of California, San Diego

**Keywords:** adverse, cutaneous, dermatitis, effect, pellet, rash, side, skin, testosterone

## Abstract

Testosterone replacement therapy is a treatment utilized for male hypogonadism. A subcutaneous testosterone pellet is a long-acting, slow-release delivery system that can be utilized as androgen replacement therapy. A 77-year-old man who was treated with testosterone pellets developed dermatitis consisting of erythematous plaques and patches on both buttocks and thighs within 28 days following the subcutaneous insertion of testosterone pellets. The skin lesions rapidly resolved with high-potency topical corticosteroid application. The same cutaneous eruption occurred with each subsequent insertion of testosterone pellets. Other cutaneous adverse events associated with testosterone pellet insertion include acne, hirsutism, and male pattern alopecia. Bleeding, bruising, fibrosis, infections, pellet extrusion, scarring, and subcutaneous nodules may also occur at the injection site. In summary, testosterone pellet-induced dermatitis is a rare adverse cutaneous event, which should be added to the list of potential testosterone pellet associated skin side effects.

## Introduction

Testosterone pellets are used in the treatment of male hypogonadism [1-3]. We describe a man who developed recurrent dermatitis associated with each subcutaneous insertion of testosterone pellets. We also review other cutaneous adverse events associated with this medication.

## Case presentation

**Figure 1:**
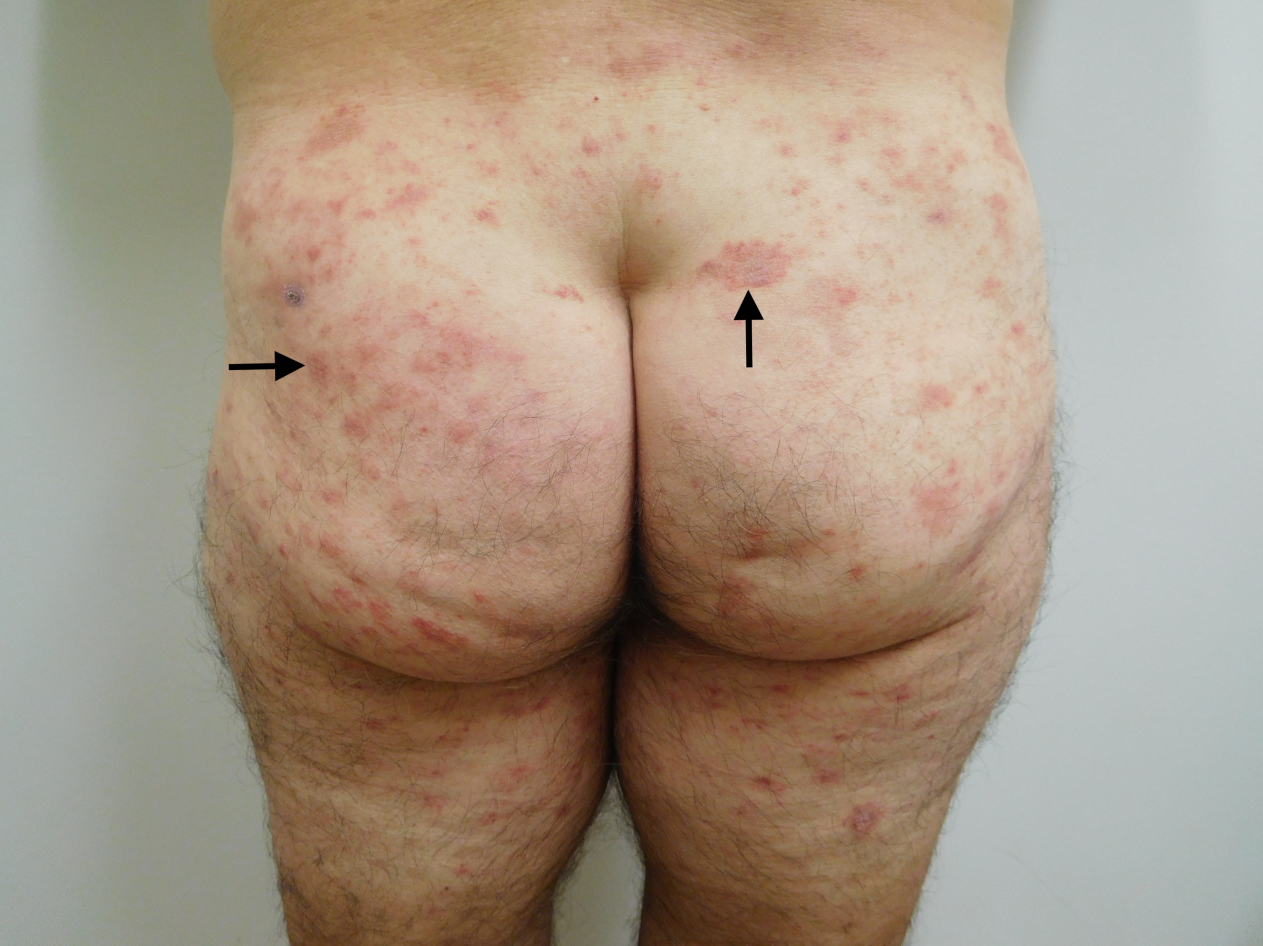
Testopel-associated dermatitis on both buttocks Cutaneous erythematous plaques and patches on the buttocks and posterior thighs (arrows show representative lesions) of a 77-year-old man, which appeared within four weeks after the insertion of 900 mg of testosterone (12 pellets).

**Figure 2:**
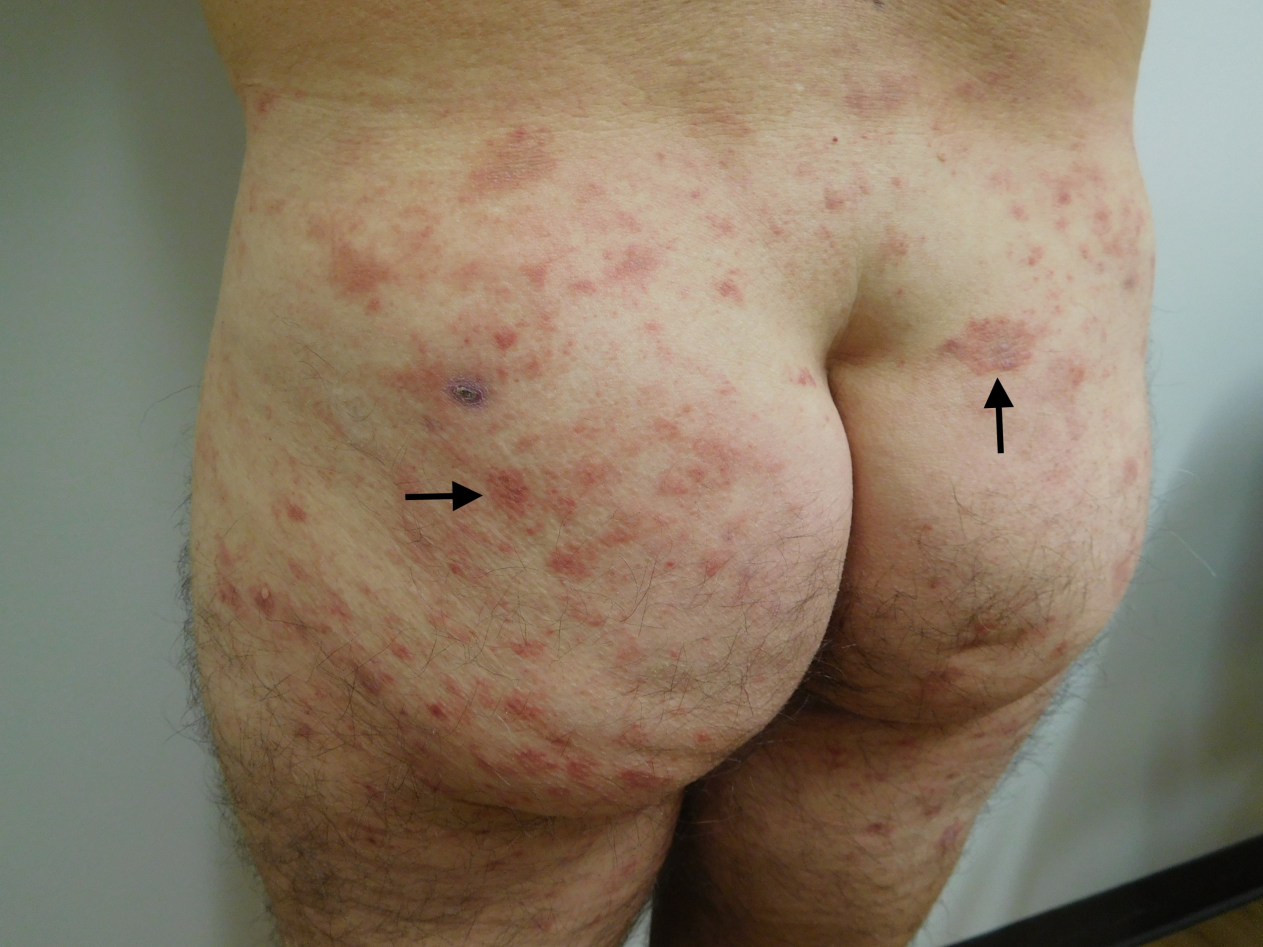
Testopel-associated dermatitis on buttocks and lateral thighs Cutaneous plaques and patches on buttocks and lateral thighs (arrows show representative lesions).

**Figure 3:**
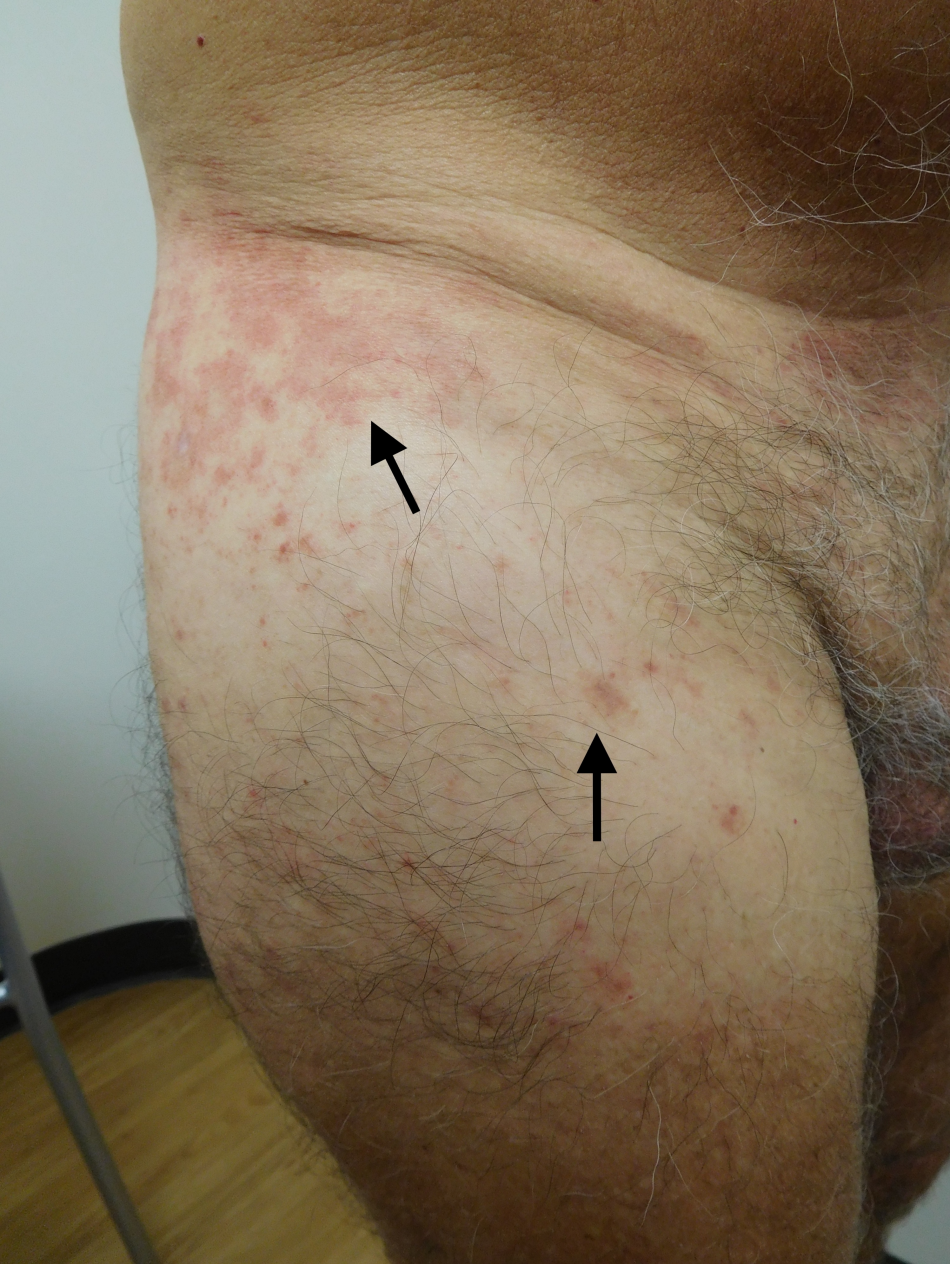
Testopel-associated dermatitis on lateral and anterior right thigh Erythematous plaques and patches (arrows show representative lesions) following insertion of 12 x 75 mg Testopel pellets.

**Figure 4:**
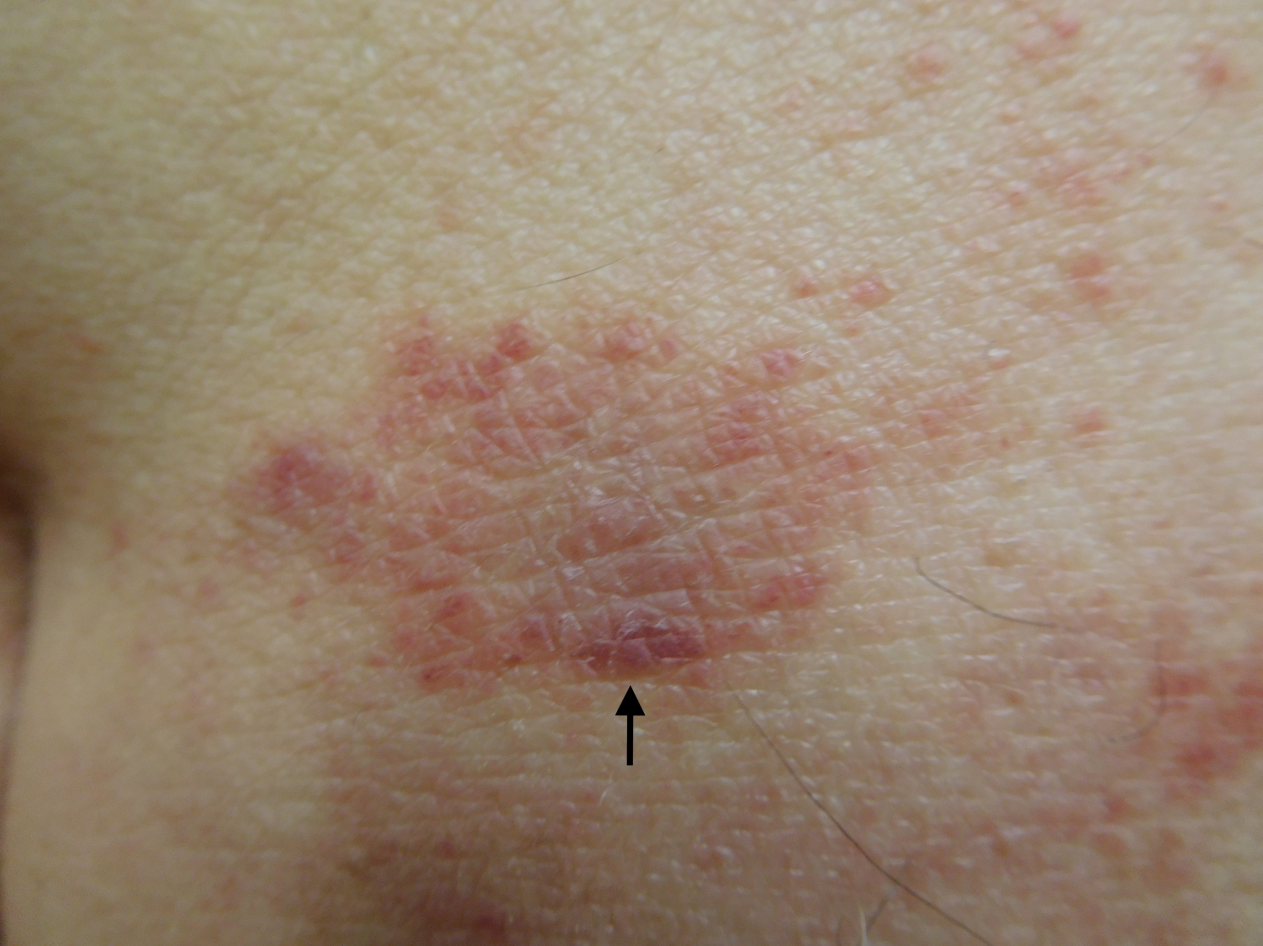
Testopel-associated dermatitis A closer view of the plaque (arrow) on the superior left buttock, which was biopsied.

A 77-year-old man presented for evaluation of a pruritic rash on both buttocks and thighs in October 2016. He has a history of hypogonadism and receives insertion of testosterone pellets (Testopel, Endo Pharmaceuticals Inc., Malvern, PA) every six months. His first insertion was 18 months earlier in April 2015; he did not develop any skin lesions after the initial insertion. However, within 28 days after each subsequent insertion (October 2015, April 2016, October 2016, and April 2017) he developed a pruritic rash on his buttocks and thighs. Skin lesions appeared within four weeks after the second treatment of 12 testosterone pellets; each pellet was 75 mg for a total dose of 900 mg. He received an intramuscular injection of triamcinolone (corticosteroid) from his primary care physician to treat the initial and subsequent episodes, which promptly treated the first and second reactions. He then presented four weeks after his fourth injection. A cutaneous examination showed erythematous plaques and patches on the buttocks. Similar lesions were on his thighs (Figures 1-4).

**Figure 5:**
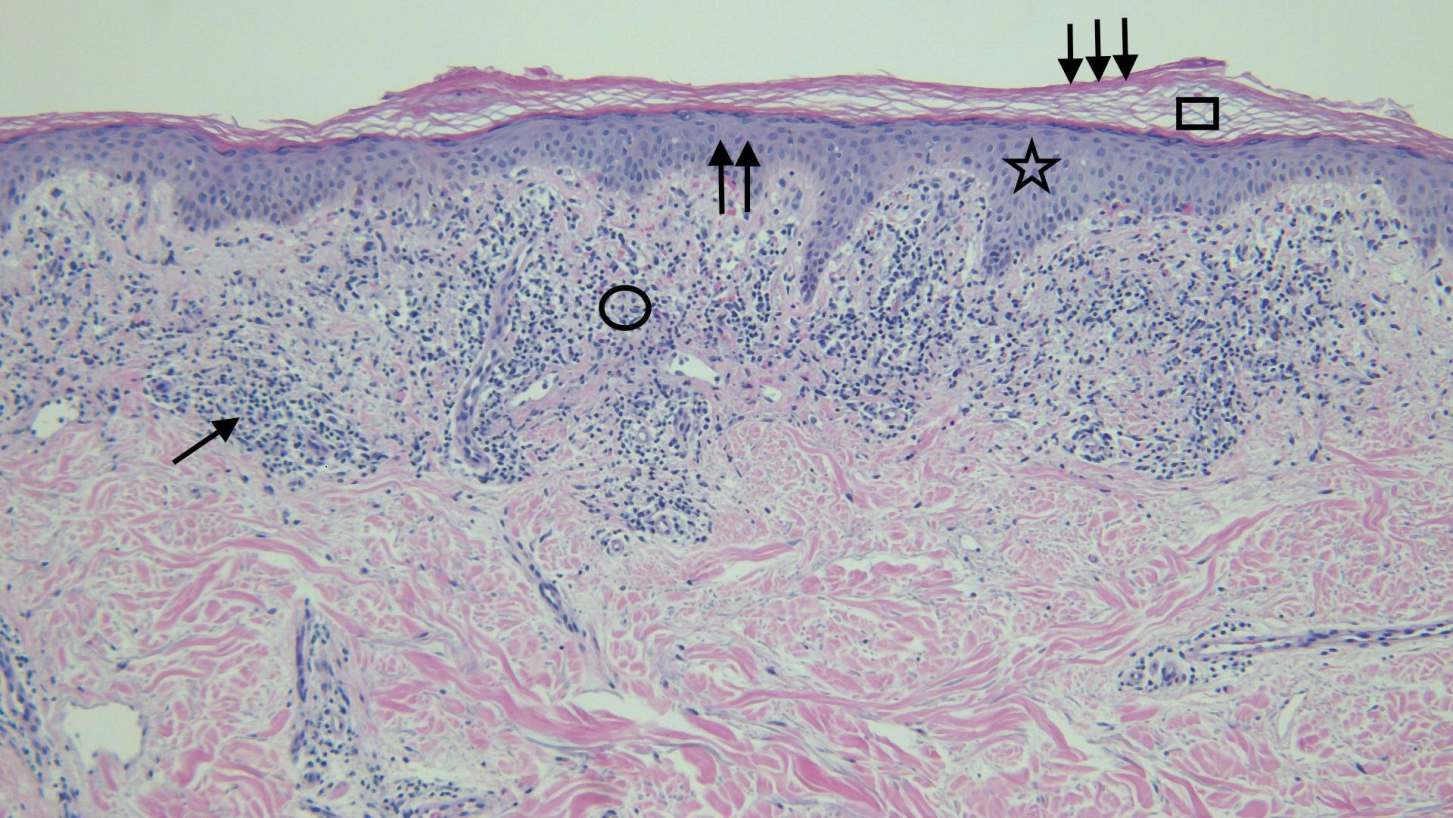
Distant view of the microscopic examination of testosterone-associated vacuolar interface dermatitis The stratum corneum (square) shows orthokeratosis (triple arrows); there is mild thickening (double arrows) of the epidermis (star). Lymphocytes (single arrow) are present in the upper dermis (circle) (hematoxylin and eosin; x = 4).

**Figure 6:**
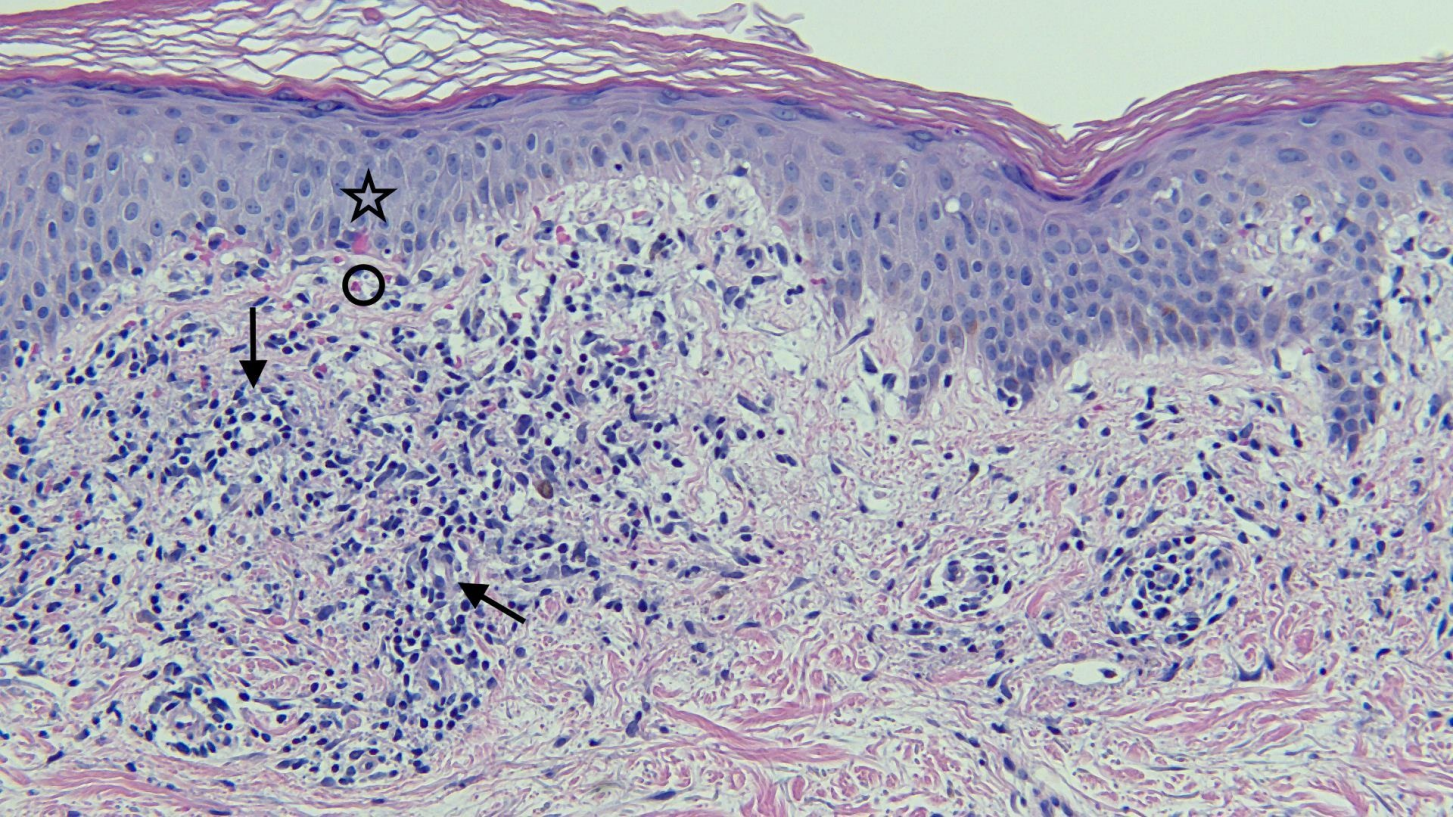
Closer view of the microscopic examination of testosterone pellet associated vacuolar interface dermatitis There is lymphocytic inflammation in the papillary dermis. The infiltrate is present around the blood vessels (arrows); it is also located at the junction between the epidermis (star) and dermis (circle) (hematoxylin and eosin; x = 20).

**Figure 7:**
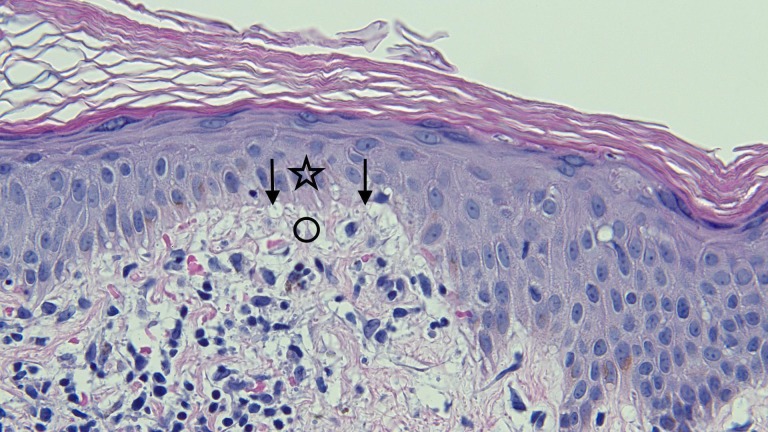
Closer view of the microscopic examination of Testopel-associated vacuolar interface dermatitis There is vacuolar alteration of the basal layer of the epidermis (arrows), and lymphocytes are present at the interface of the epidermis (star) and dermis (circle) (hematoxylin and eosin; x = 40).

A microscopic examination of the skin biopsy showed orthokeratosis and mild acanthosis of the epidermis. Neither spongiosis nor elongation of the rete ridges were prominent. There was vacuolar alteration of the epidermal basal layer. An inflammatory infiltrate of lymphocytes was present not only around the blood vessels in the upper dermis, but also at the junctions between the epidermis and the dermis. In summary, the changes are those of a vacuolar interface dermatitis (Figures 5-7).

Correlation of history, clinical morphology, and pathology established the diagnosis of testosterone pellet associated dermatitis. Topical treatment with a high potency corticosteroid cream (clobetasol propionate 0.05% twice daily) was initiated and the skin lesions completely resolved within a week.

**Figure 8:**
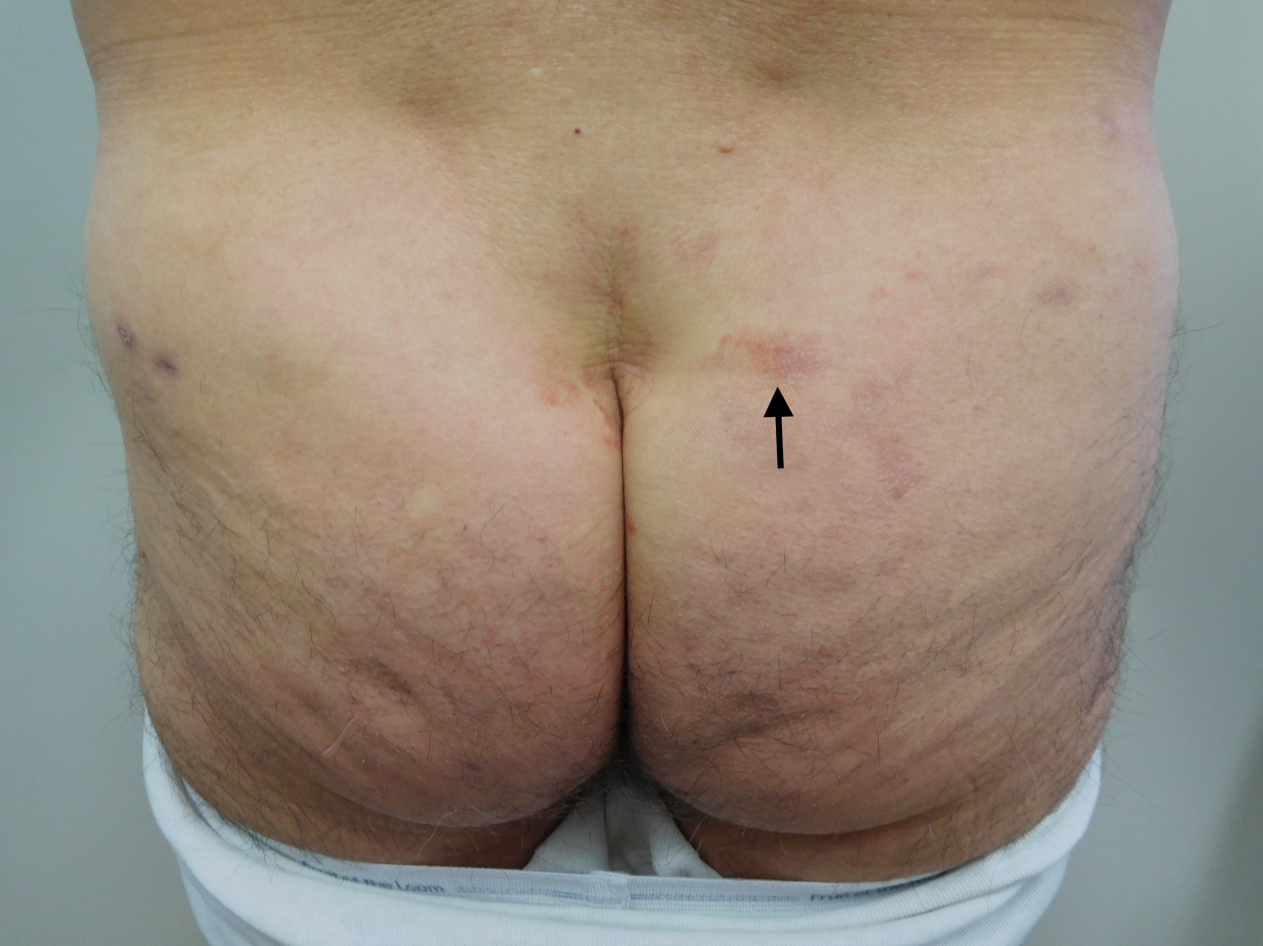
Recurrent Testopel-associated dermatitis The patient developed cutaneous lesions (arrow) similar to his prior episode of dermatitis, however, with lesser severity, at the same locations on his buttocks and thighs after receiving a decreased dose of 750 mg testosterone (10 x 75 mg testosterone pellets).

**Figure 9:**
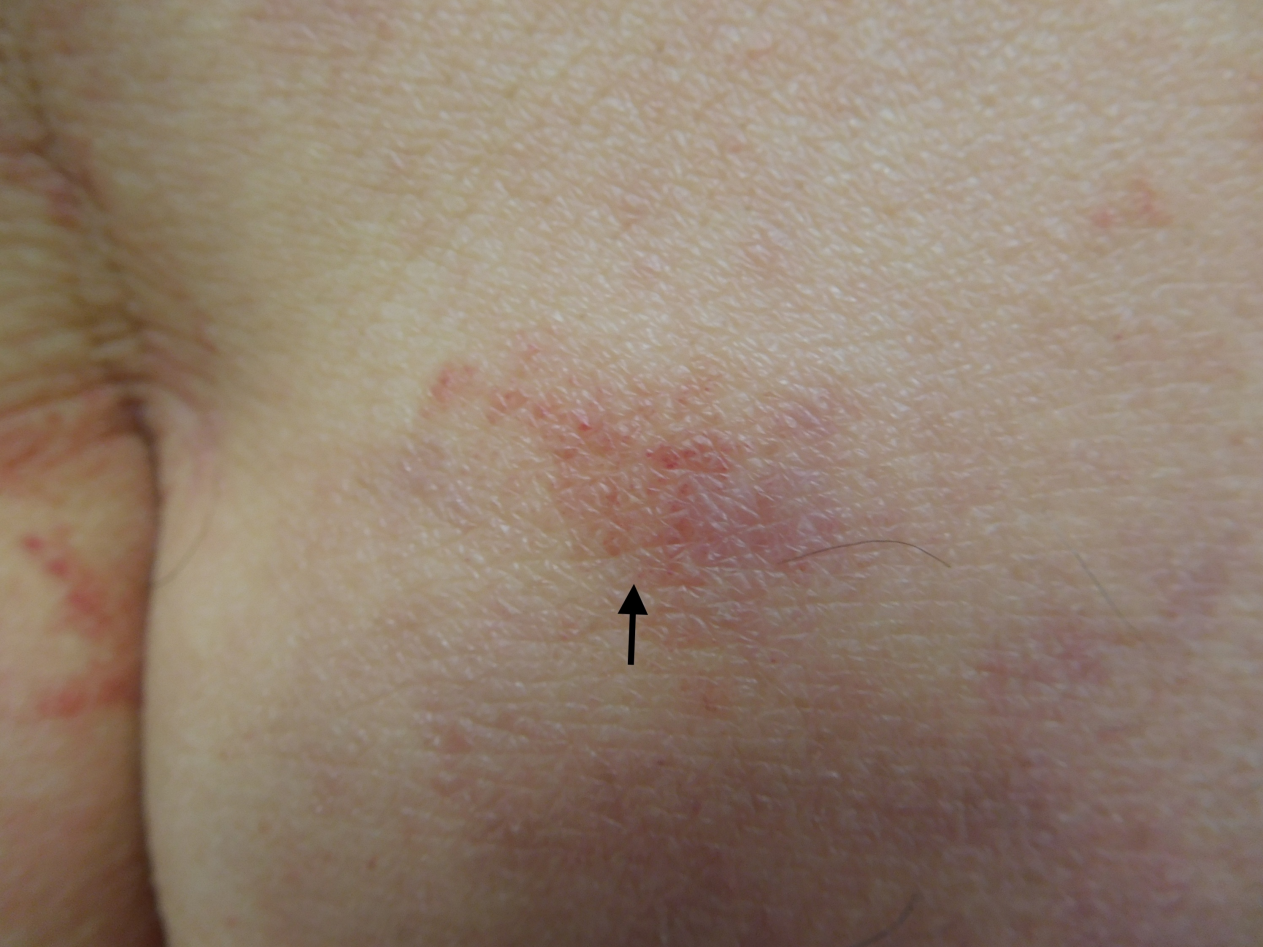
Recurrent Testopel-associated dermatitis A closer view of the plaque (arrow) on the right buttock, which developed after the patient received 750 mg testosterone. The plaque was biopsied.

The likelihood of re-occurrence of the dermatitis with subsequent testosterone pellet therapy was discussed with the patient. However, he insisted on continuing treatment of his hypogonadism with the same medication. A decreased dose of 750 mg (ten 75 mg testosterone pellets) was inserted six months later in April 2017. Within 28 days, he developed similar cutaneous plaques and patches, of lesser severity, on the same location of his buttocks and thighs (Figures 8-9).

The biopsy of the new skin lesion showed the same pathologic changes as his previous skin biopsy. The skin lesions completely resolved within a week after initiating treatment with clobetasol propionate 0.05% cream.

## Discussion

Male hypogonadism occurs when the body is not able to produce or keep testosterone at physiological level [2-3]. It can adversely affect many tissues and organs. Some of the symptoms of male hypogonadism include decreased lean body mass and strength, increased adipose tissue mass, mood disorders, and sexual dysfunction, especially diminished libido [1-3].

Male hypogonadism is frequently associated with aging [2, 4]. It typically affects middle-aged and older-aged men [4]. The diagnosis of male hypogonadism is established by observing persistent signs and symptoms of low testosterone accompanied with a low total serum testosterone level [2-3]. However, there is an increased prevalence of low testosterone and hypogonadism in men with chronic obstructive pulmonary disease, end-stage renal disease, human immunodeficiency virus (HIV) infection, and type 2 diabetes mellitus [2].

Testosterone is the main treatment for male hypogonadism. It is available in several forms: buccal tablets, oral capsules or tablets, intramuscular injections, and transdermal patches and gels [1-3]. In addition, male hypogonadism can also be treated by periodically administrating subcutaneous testosterone pellets.

Testosterone pellets are usually well tolerated. Adverse cutaneous events and other drug-associated side effects are listed in Table 1 [1, 3, 5-10]. While acne, alopecia (male pattern), and hirsutism are common skin side effects, other cutaneous adverse events like bleeding, bruising, fibrosis, infections, pellet extrusion, scarring, and subcutaneous nodules can occur at the implantation site. To the best of our knowledge, our patient is the first man described with testosterone pellet induced dermatitis.

**Table 1:** Adverse cutaneous events and other testosterone drug associated side effects

Location	Side Effects
Cardiovascular	Myocardial infarction, stroke
Endocrine and urogenital	Gynecomastia, excessive frequency and duration of penile erections
Fluid and electrolytes disturbances	Retention of calcium, chloride, inorganic phosphates, potassium, sodium and water
Gastrointestinal	Alteration in liver function tests, cholestatic jaundice, nausea, rarely hepatocellular neoplasms and peliosis hepatis
Hematologic	Bleeding in patients on concomitant anticoagulant therapy, suppression of clotting factors II, V, VII, and X
Implantation site events	Bleeding, bruising, fibrosis, infection, pellet extrusion, scarring, subcutaneous nodules
Metabolic	Increased serum cholesterol
Miscellaneous	Rarely anaphylactoid reactions
Nervous system	Anxiety, depression, generalized paresthesia, headaches, increased or decreased libido
Skin and appendages	Acne, alopecia (male pattern), hirsutism
Vascular disorders	Venous thromboembolism

Testosterone-induced dermatitis is a rare cutaneous side effect. Our patient developed biopsy-proven vacuolar interface dermatitis after each administration of the drug. The occurrence of testosterone pellet associated dermatitis is not a drug-limiting side effect; the severity and extent of dermatitis was significantly diminished by decreasing the dose of testosterone implanted.

## Conclusions

Subcutaneous testosterone pellets are a form of androgen replacement therapy that is used to treat male hypogonadism. Several cutaneous side effects have been described in patients receiving this medication, including acne, male pattern alopecia, hirsutism, and implantation site events. Recurrent dermatitis can be added to this list of potential adverse skin events in men receiving testosterone pellet insertions.
